# 

**Published:** 1898-10

**Authors:** 


### REPRINTS RECEIVED.

The Conservative Treatment of Fibroid Tumors by Myomectomy; The Conservative Treatment of Pelvic Suppuration of Puerperal Origin. By Charles P. Noble, M.D., of Philadelphia.

Report of the Kensington Hospital for Women, 136 Diamond Street, Philadelphia.

Advance in the Domain of Preventive Medicine. By J. M. B. Carter, M.D., Waukegan, Ill.

Principal Poisonous Plants of the United States. By V. K. Chestnut. Produced from the United States Department of Agriculture, Division of Botany.

Post-Operative Insanity. By R. Harvey Reed, Rock Springs, Wyoming.

Michigan Monthly Bulletin of Vital Statistics, August.

From Dr. E. C. Ellett, Memphis, Tenn.:

1: Acute Inflammation of the Middle Ear.
2. A Case of Tenonitis.
3. The Determining Cause of the Site of Ulcers on the Nasal Septum.
4. Hemorrhagic Glaucoma. Report of a Case with Microphotographs.

